# Atopic dermatitis pediatric patients show high rates of nasal and intestinal colonization by methicillin-resistant *Staphylococcus aureus* and coagulase-negative staphylococci

**DOI:** 10.1186/s12866-023-03165-5

**Published:** 2024-01-29

**Authors:** Mariana Fernandes Augusto de Oliveira, Daiane Bitencourt Agne, Ludmila Sento Sé Bastos, Laura Maria Andrade de Oliveira, Simone Saintive, Ekaterini Simoes Goudouris, Evandro Alves do Prado, Henrique Fragoso dos Santos, Raphael da Silva Pereira, Fernanda Sampaio Cavalcante, Dennis de Carvalho Ferreira, Kátia Regina Netto dos Santos

**Affiliations:** 1 Laboratório de Infecção Hospitalar, Departamento de Microbiologia Médica, Instituto de Microbiologia Paulo de Góes, Universidade Federal do Rio de Janeiro, CCS, Bloco I, Sala I2-010, UFRJ. Cidade Universitária, Rio de Janeiro, RJ Brasil CEP: 21941-590; 2 Laboratório de Cocos Patogênicos e Microbiota, Departamento de Microbiologia Médica, Instituto de Microbiologia Paulo de Góes, Universidade Federal do Rio de Janeiro, Rio de Janeiro, Brasil; 3 Serviço de Dermatologia Pediátrica, Instituto de Puericultura e Pediatria Martagão Gesteira, Universidade Federal do Rio de Janeiro, Rio de Janeiro, Brasil; 4 Serviço de Imunologia Pediátrica, Instituto de Puericultura e Pediatria Martagão Gesteira, Universidade Federal do Rio de Janeiro, Rio de Janeiro, Brasil; 5 Departamento de Biologia Marinha, Universidade Federal Fluminense, Rio de Janeiro, Brasil; 6 Laboratório de Biotecnologia e Ecologia Microbiana, Departamento de Microbiologia Geral, Instituto de Microbiologia Paulo de Góes, Universidade Federal do Rio de Janeiro, Rio de Janeiro, Brasil; 7 Departamento de Clínica Médica, Instituto de Ciências Médicas, Centro Multidisciplinar de Macaé, Universidade Federal do Rio de Janeiro, Macaé, Brasil; 8 Faculdade de Odontologia, Universidade Veiga de Almeida, Rio de Janeiro, Brasil; 9 Faculdade de Odontologia, Universidade Estácio de Sá, Rio de Janeiro, Brasil; 10 Universidade do Estado do Rio de Janeiro, Rio de Janeiro, Brasil

**Keywords:** Atopic dermatitis, Nasal colonization, Intestinal colonization, *Staphylococcus* spp, Antimicrobial resistance, Clonality, Gut bacterial community

## Abstract

**Background:**

Atopic dermatitis (AD) patients have high rates of colonization by *Staphylococcus aureus*, which has been associated with worsening of the disease. This study characterized *Staphylococcu*s spp isolates recovered from nares and feces of pediatric patients with AD in relation to antimicrobial susceptibility, staphylococcal cassette chromosome *mec* (SCC*mec*) type, presence of *pvl* genes and clonality. Besides, gut bacterial community profiles were compared with those of children without AD.

**Results:**

All 55 AD patients evaluated had colonization by *Staphylococcus* spp. Fifty-three (96.4%) patients had colonization in both clinical sites, whereas one patient each was not colonize in the nares or gut. *Staphylococcus aureus* was identified in the nostrils and feces of 45 (81.8%) and 39 (70.9%) patients, respectively. Methicillin-resistant *Staphylococcus* spp. isolates were found in 70.9% of the patients, and 24 (43.6%) had methicillin-resistant *S. aureus* (MRSA). *S. aureus* (55.6%) and *S. epidermidis* (26.5%) were the major species found. The prevalent lineages of *S. aureus* were USA800/SCC*mec*IV (47.6%) and USA1100/SCC*mec*IV (21.4%), and 61.9% of the evaluated patients had the same genotype in both sites. Additionally, gut bacterial profile of AD patients exhibits greater dissimilarity from the control group than it does among varying severities of AD.

**Conclusions:**

High rates of nasal and intestinal colonization by *S. aureus* and methicillin-resistant staphylococci isolates were found in AD patients. Besides, gut bacterial profiles of AD patients were distinctly different from those of the control group, emphasizing the importance of monitoring *S. aureus* colonization and gut microbiome composition in AD patients.

**Supplementary Information:**

The online version contains supplementary material available at 10.1186/s12866-023-03165-5.

## Introduction

Atopic Dermatitis (AD) is a chronic and relapsing skin disorder characterized by highly pruritic lesions and age-depending distribution [1, 2]. The disease affects approximately 10-20% of children and 1-3% of adults in developed countries [3, 4] and is classified into mild (SCORAD < 25), moderate (25-50) and severe (>50) categories based on the SCORAD (scoring atopic dermatitis) index [5]. Although the exact pathogenesis of AD is unclear, disruption of the epithelial barrier, immune dysregulation, environmental exposure, and skin/gut microbiome dysbiosis are thought to be involved [6–8].

*Staphylococcus aureus* is an opportunistic pathogen associated with mild to life-threatening infections, and it can colonize skin and mucous membranes such as anterior nares and the intestinal tract [9–12]. In patients with AD, *S. aureus* has been implicated in the worsening of skin lesions and its colonization rates in the nasal and skin areas can range from 30 to 90% [13–15]. Methicillin-resistant *S. aureus* (MRSA) isolates are commonly detected in AD patients and are associated with well-established community clones carrying SCC*mec* types IV or V [15–17]. However, the prevalence of intestinal colonization by *S. aureus* in AD patients remains unknown. To address this gap in knowledge, the current study aimed to isolate and identify *Staphylococcus* species from nares and feces of AD pediatric patients attending in a reference center in Rio de Janeiro, Brazil and characterize their methicillin resistance. The study also evaluated aspects associated with the virulence and clonality of *S. aureus* and compared the profiles of gut bacterial communities between AD patients and children without the disease.

## Methods

### Study population

A cross-sectional clinical and laboratory study was conducted on pediatric patients with AD who were attended at a Dermatology Service of a public pediatric hospital in Rio de Janeiro, between November 2015 to July 2018. Patients with ages ranging from two to ten years old, diagnosed with AD and classified by SCORAD were included.

Exclusion criteria were patients with another chronic dermatological disease, previous hospitalizations within the past six months, and diarrhea episodes at the time of the fecal sample collection. For comparison of gut bacterial profiles, a control group composed of nine children without the disease, aged between two and ten years old, was also included.

### Clinical specimens and bacterial isolates

Nasal swab and fecal samples were collected from each AD patient. Approximately 0.4 g of fecal sample was emulsified in 1 mL of TE buffer (30 mM Tris-HCL and 1 mM EDTA, pH 8). Both specimens were cultured on Mannitol salt agar (BD, New Jersey, USA) and incubated at 35 ºC for 48h. Colonies with distinct characteristics were selected from the plates of each clinical sample. Bacterial identification was performed using matrix-assisted laser desorption ionization time-of-flight mass spectrometry (MALDI-TOF/MS) (Bruker Daltonics, Massachusetts, USA) with the software MALDI Biotyper version 7.0 (Bruker Daltonics). The same procedure was performed for the control group.

### Antimicrobial susceptibility tests

To evaluate the antibiotic susceptibility of the *Staphylococcus* isolates, disk-diffusion test was performed based on the guidelines of CLSI (2019) [18]. In addition to methicillin (cefoxitin disk used), *S. aureus* isolates were tested for susceptibility to ciprofloxacin, clindamycin, erythromycin, gentamicin, mupirocin, penicillin, rifampicin, trimethoprim-sulfamethoxazole (TMP/STX) and tetracycline (Oxoid, Cambrigde, UK) [18]. For *S. aureus* isolates that were classified as resistant to mupirocin by the disk-diffusion method, a Minimum Inhibitory Concentration (MIC) determination was performed using Etest® (BioMérieux, North Carolina, USA). Multidrug resistance (MDR) was defined as the presence of resistance to at least three classes of antimicrobials, except penicillin. The disk-diffusion test and Etest® were quality-controlled using *S. aureus* ATCC 25923 and ATCC 29213 as reference strains, respectively.

### SCC*mec* typing and *pvl* genes detection

For all *S. aureus* and *S. epidermidis* isolates, bacterial DNA was extracted through enzymatic lysis [19]. SCC*mec* typing, according to Milheriço et al. (2007) [20], was performed on MRSA isolates while methicillin-resistant *S. epidermidis* (MRSE) isolates were evaluated following the protocol by Kondo and colleagues (2007) [21]. Positive controls for *Staphylococcus* strains are described in Salgueiro et al. (2009) [22]. To detect PVL-encoding genes, all *S. aureus* isolates were screened using the method described by Lina and colleagues (1999) [23], and the 526a isolate was used as positive control [24].

### Genotyping tests

*Staphylococcus aureu*s isolates obtained simultaneously from nares and feces of patients colonized by MRSA in at least one clinical site were subjected to pulsed-field gel electrophoresis (PFGE). Genomic DNA was digested with *Sma*I (New England Biolabs, Massachusets, USA) and submitted to a CHEF-DRIII system (Bio-Rad, California, USA), as described previously [25]. The PFGE profiles were compared using the unweighted pair-group method arithmetic mean (UPGMA) clustering analysis with the Dice correlation coefficient. Isolates with four or fewer bands of difference and a minimum of 80% similarity were considered to belong to the same genotype [26]. The clonal lineages were defined by comparison with national and international previously identified clones [27, 28].

To complement the clonal identification, some isolates were submitted to *spa* typing, according to Larsen et al. (2008) [29]. After detecting the *spa* gene by PCR the amplicons were purified using the GTX PCR and band purification (GE Healthcare, Illinois, USA). The DNA was sequenced using MegaBACE 1000 system. The obtained sequences were analyzed using BioEdit software 7.2 and assigned to a specific *spa* type using the ST *spa*Typer server database (https://spatyper.fortinbras.us/). The *spa* type DNA sequences obtained are available in a txt.file (Supplementary material).

### Analysis of intestinal bacterial profiles by DGGE

Denaturing Gradient Gel Electrophoresis (DGGE) was utilized to analyze fecal samples from both AD patients and healthy children. DNA was obtained by Xpedition™ Soil/Fecal DNA MiniPrep Kit (Zymo, California, USA), following the manufacturer’s instructions. The concentration of DNA was measured using NanoVue Plus (GE Healthcare Life Science, Illinois, USA) and stored at -20°C.

PCR amplification of the 16S rRNA gene was carried out using primer U968f-GC1 5’ AAC GCG AAG AAC CTT AC 3’, with a GC-clamp at the 5’end and L1401r 5’GCG TGT GTA CAA GAC CC 3’, which is homologous to *Escherichia coli* 16S rRNA [30]. DGGE analysis was performed using the Dcode, Universal Mutation Detection System (Bio-Rad, California, USA). The amplified products were loaded onto an 8% polyacrylamide (Sigma-Aldrich Chemical Company, Wisconsin, USA) gel with a denaturing gradient set as 46.5-60% urea/formamide (Promega, Wisconsin, USA). Electrophoresis was carried out in 1X TAE buffer at 50V and 60°C for 17 hours. The DGGE gel was stained with SYBR Gold (Invitrogen, Massachusets, USA) and visualized using a Storm 860 Imaging System (GE Healthcare, Illinois, USA). DGGE gel profiles were analyzed using BioNumerics software 7.2 (Applied Maths, Ghent, Belgium). Result patters were compared using Dice similarity coefficient and clustered by unweighted pair group method with arithmetic (UPGMA). To analyze the grouping patterns of the samples, principal component analysis (PCA) was employed. The first two principal components, namely axis 1 and axis 2, were retained for interpretation, with axis 1 explaining the majority of the variability in the data, and axis 2 explaining the second largest portion of variability. The input data for the analysis consisted of matrices containing the band intensities of the analyzed samples. In addition, diversity measures were computed through the following methods: Richness (S) was determined by counting the number of bands present in each lane. Additionally, the diversity of each sample was evaluated using the Shannon-Weaver diversity index (H’), which was computed through the formula H’ = –Σ (Pi × log Pi). In this equation, Pi represents the probability of the importance of each peak within the densitometric profile, which was obtained by dividing the height of each peak (ni) by the sum of all peak heights (N). Above statistical analyses were done using Canoco (Canoco 4.5, Biometris, Wageninge, NE) software package [31].

### Statistical analysis

All data were analyzed using the SPSS (SPSS Statistics v. 19.0; IBM Brazil, São Paulo, Brazil). Kruskal Wallis and Mann-Whitney U tests were performed to verify the difference between groups regarding DGGE to evaluate bacterial richness and diversity. Besides, the exact Fisher’s test and chi-square test were used to compare data. Significance was established at 5% (*p*-value <0.05).

## Results

### Characteristics of pediatric patients

In this study, a total of 55 AD pediatric patients diagnosed with AD were enrolled, with a majority of them being male (52.7%, 29/55) and having a median age of 5.7 years old. Based on the SCORAD index, 13 (23.6%) patients had mild AD, 25 (45.5%) had moderate, and 17 (30.9%) had severe. The control group consisted of 9 healthy children, with a higher portion of males (66.7%, 6/9) and median age of 5.9 years old (Supplementary Table 1).

### Bacterial isolates

All 55 AD patients in the study presented *Staphylococcus* spp. colonization, 53 patients (96.4%) showed colonization in both clinical sites, whereas one patient each was not colonized in the nares or gut.

**Fig. 1:**
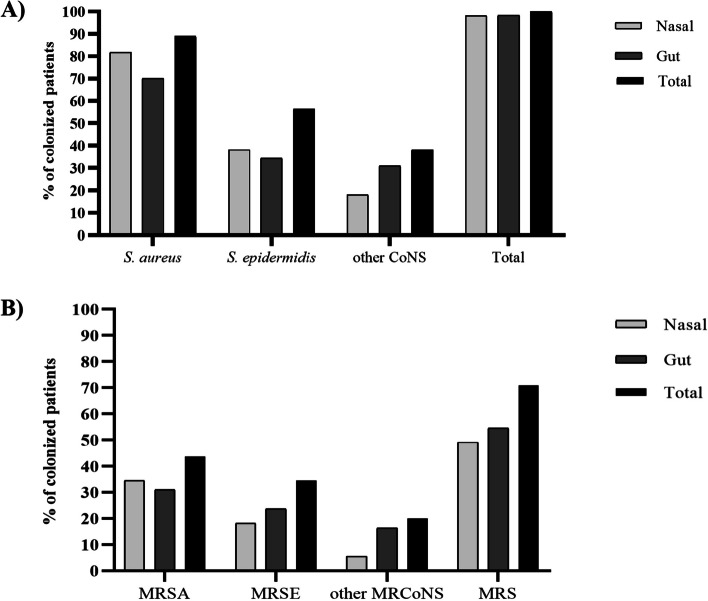
Nasal and gut colonization by *Staphylococcus* spp in 55 patients with atopic dermatitis. **A** Patients colonized in nares and/or gut by *Staphylococcus* spp isolates. **B** Patients colonized in nares and/or gut by methicillin-resistant *Staphylococcus* spp isolates. CoNS – coagulase-negative *Staphylococcus*; MRSA – methicillin-resistant *S.* *aureus*; MRSE - methicillin-resistant *S. epidermidis*; MRCoNS - methicillin-resistant CoNS; MRS – methicillin-resistant *Staphylococcus* spp.

*S. aureus* was found in 49 (89.1%) patients, with 45 (81.8%, 45/55) and 39 (70.9%, 39/55) of them presenting the microorganism in nasal and/or fecal samples, respectively (Fig. 1). It is noteworthy the fact that the pathogen was the unique staphylococcal species found in nares and/or feces of 25 (45.5%) and 22 (40%) patients, respectively. Besides, 14 (25.5%) patients showed exclusive colonization by *S. aureus* in both niches (Supplementary Table 1).

Coagulase-negative staphylococci (CoN*S*) isolates were found in 40 (72.7%) patients, with 29 (52.7%) and 32 (58.2%) of patients presenting these microorganisms in the nares and/or feces, respectively. *S. epidermidis* was the most frequent CoNS species (56.4%, 31/55) in AD patients, being 38.2% in nares and 32.7% in feces (Fig. 1). The coexistence of *S. aureus* and *S. epidermidis* in the nasal niche was observed in 16 (29.1%) patients, while both species were found in feces of 13 (23.6%) patients. Other CoNS species were present in 21 (38.2%) patients (Fig. 1, Supplementary Table 1).

**Fig. 2:**
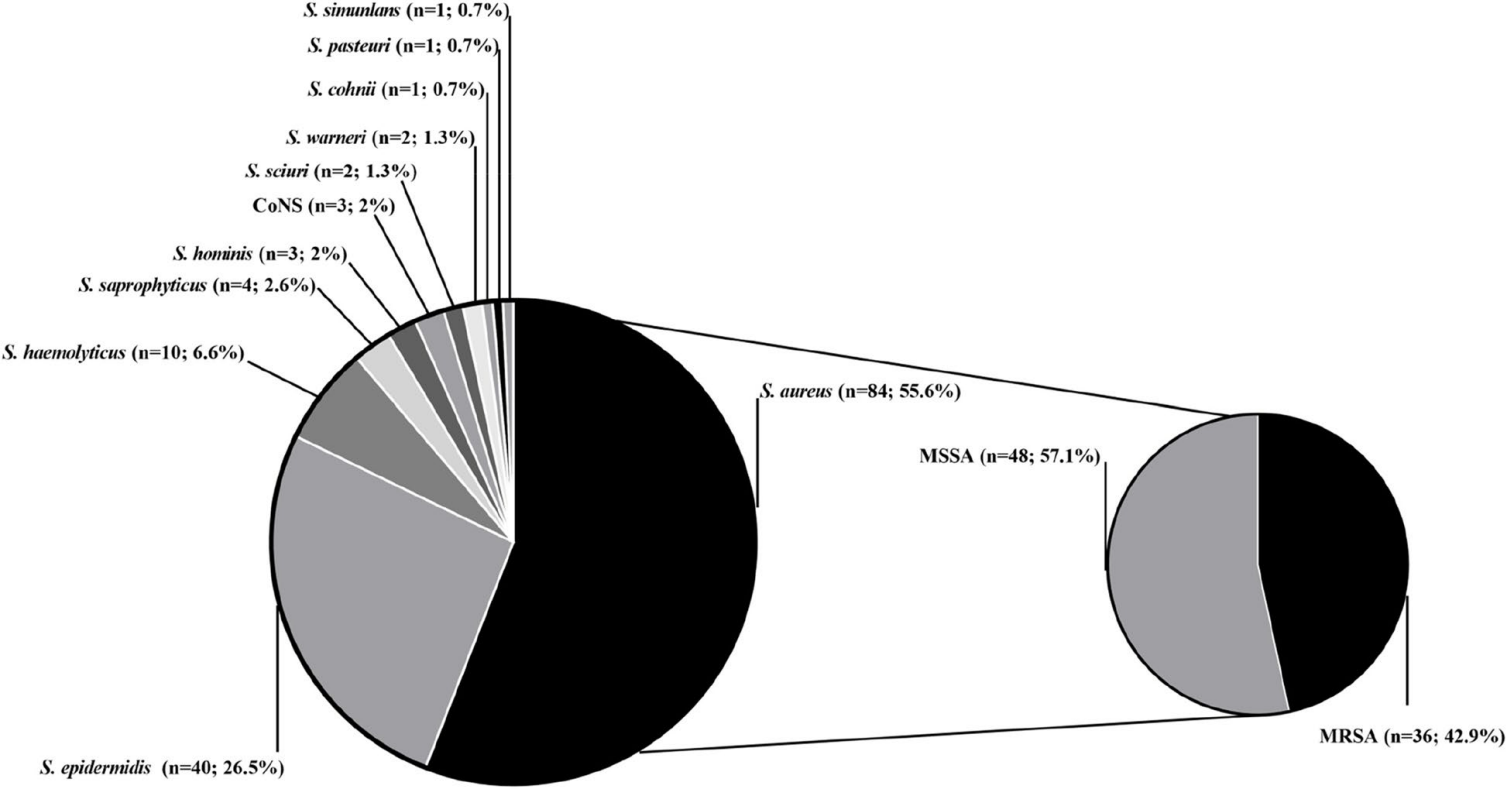
Distribution of *Staphylococcus* species among 151 isolates from nares and/or feces of atopic dermatitis pediatric patients. n - number of isolates; Three (2%) CoNS isolates were not identified at species level; MRSA – Methicillin-resistant *S. aureus*; MSSA – Methicillin-sensitive *S. aureus*.

Overall, 151 staphylococcal isolates were identified from 110 clinical specimens obtained from AD patients, with 75 isolates from nasal swabs and 76 from fecal samples. Most isolates (55.6%, 84/151) were identified as *S. aureus,* while *S. epidermidis* was the most frequent CoNS species (26.5%, 40/151) followed by *Staphylococcus haemolyticus* (6.6%, 10/151). Three (2%) CoN*S* isolates could not be identified to the species level (Fig. 2).

For comparison of gut bacterial profiles, fecal samples from a control group of nine children were analyzed. Among them, eight (88.9%) individuals showed *Staphylococcus spp.* in feces, being 14 *Staphylococcus* spp isolates recovered. *S. aureus* (21.4%, 3/14), *S. epidermidis* (21.4%, 3/14) and *S. haemolyticus* (21.4%, 3/14) were the main species found. Besides, colonization by MRSA isolates was not described in the control group (Supplementary Table 1).

### Antimicrobial susceptibility, SCC*mec *types, and *pvl* genes

Methicillin-resistant *Staphylococcus* spp. isolates were found in 39 (70.9%) patients. Twenty-four (43.6%) patients had methicillin-resistant *S. aureus* (MRSA) isolates, 19 (34.5%) in nares and 17 (31%) in gut (Fig. 1). Nineteen (34.5%) patients were colonized exclusively in the nares and/or the gut by MRSA isolates. Other 13 patients also had *S. aureus* at both sites, but none were MRSA (supplementary Table 1). There was no correlation between MRSA colonization and SCORAD index classification.

Methicillin-resistant CoNS (MRCoNS) isolates were found in 25 (45.5%) patients, and 19 (34.5%) had MRSE isolates, being 10 (18.2%) and 13 (23.6%) in the nares and the gut, respectively. Other MRCoNS, non-*S. epidermidis* were found in 20% of patients (Fig. 1).

A total of 84 *S. aureus* isolates (45 from nares and 39 from gut) were recovered in the study and the antimicrobial resistance has been found to penicillin (95.2%), erythromycin (42.9%), methicillin (42.9%), clindamycin (23.8%), gentamicin (17.9%), tetracycline (6%), mupirocin (3.6%) and trimethoprim-sulfamethoxazole (2.4%) (Supplementary Table 2). Three MRSA isolates (two nasals and one fecal) were resistant to mupirocin (MIC values of 8 and 64 mg/L [patient 34] and ≥ 1024 mg/L [patient 18]). All isolates were susceptible to ciprofloxacin and rifampicin. Besides, 16.7% of MDR *S. aureus* isolates were detected. Among 36 MRSA isolates detected the SCC*mec* typing showed that most of them carried the SCC*mec* type IV (91.7%, 33/36) followed by SCC*mec* type III (5.6%, 2/36) and SCC*mec* type V (2.8, 1/36) (Supplementary Table 1). Among the 34 MRCoNS isolates, 52.2% carried the SCC*mec* type V, 17.4% the SCC*mec* type IV, and 30.4% had non-typable SCC*mec*s (supplementary Table 1).

PVL-encoding genes were found in 13 (15.5%) *S. aureus* isolates, with 10 being MRSA and three MSSA. These *pvl* positive isolates were present in 10 (18.2%) patients and were more prevalent in the severe AD group (*p*-value <0.05).

### PFGE and spa typing analysis

To verify a possible relationship between *S. aureus* isolates colonizing both clinical sites a total of 42 isolates (33 MRSA and 9 MSSA) recovered from 21 AD patients who had at least one isolate MRSA were evaluated by genotypic methods. Clinical and microbiological characteristics associated to the 21 AD patients were described in Table 1. Only three MRSA isolates from three patients (6, 16 and 49) were not evaluated by PFGE because they had colonization by *S. aureus* in only one clinical site. The prevalent lineages were USA800/SCC*mec*IV (47.6%; 20/42), USA1100/SCC*mec*IV (21.4%; 9/42), USA400/SCC*mec*IV (7.1%; 3/42), BEC/SCC*mec*III (4.7%; 2/42) and USA600/SCC*mec*IV (2.4%; 1/42). Among 27 isolates evaluated by *spa* typing 13 types were identified: t002 and t067 in USA800; t318, t6726, t1130 and t1154 in USA1100; t189 in USA400; t037 in BEC; t5693 in USA600, and the random *spa* types t065, t180, t5189 and t1451.

**Table 1:**
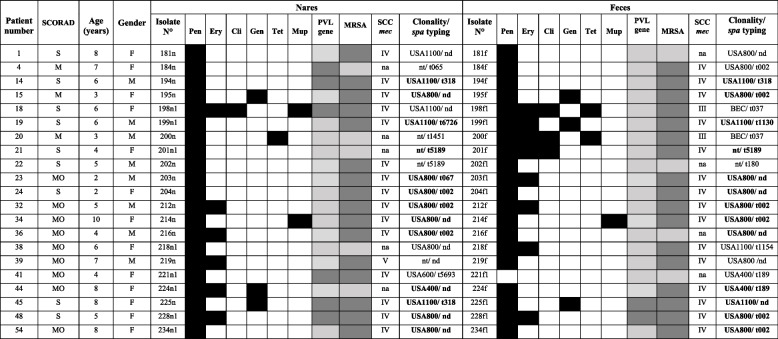
Clinical and microbiological characteristics associated to *Staphylococcus aureus* isolates from both nares and feces of 21 AD pediatric patients who had at least one MRSA colonization

-Resistant;  - Sensitive;  - Positive;  - Negative; *SCORAD* Scoring atopic dermatitis, *M* mild, *MO* Moderate, *S* Severe, *F* Female, *M* Male, *Pen* Penicillin, *Ery* Erythromycin, *Cli* Clindamycin, *Gen* Gentamycin, *Tet* Tetracycline, *Mup* Mupirocin, *PVL* Panton-Valentine leukocidin, *SCCmec* Staphylococcal chromosome cassete *mec*, *spa* Gene coding protein A, *BEC* Brazilian endemic clone, *nt *Not typable, *nd* Not determined, *na *Not aplicable (MSSA isolate); Bold – isolates belonging to a same clonal lineage and recovered from a same patient

Thirteen (61.9%, 13/21) patients presented genetically related isolates in their nostrils and feces, according to the methods used. Among them, 61.5% (8/13) presented USA800/ST5/SCC*mec*IV and 23.1% (3/13) had USA1100/ST30/SCC*mec*IV (Table 1).

### Molecular fingerprint of gut microbiome

We assessed the gut bacterial profiles of 45 AD patients and nine control children using DGGE. Chao estimate was higher in the moderate AD group (mean: 22.04/SD: 5.58), compared to the mild (mean: 18/SD: 3.04) and severe (mean: 16.21/SD: 3.62) AD groups, as well as the control group (mean: 17/SD: 2.64) (*p*-value <0.05, Mann Whitney U test). We also evaluated Shannon estimate for bacterial diversity and a significant difference was observed between the evaluated groups (*p*<0.05, Kruskal Wallis test). Besides, when the different groups were compared, those of severe (mean: 1.98/SD:0.96) and moderate (mean:1.05/SD:0.47) AD were statistically different from the control group (mean: 0.94/SD:0.01) (*p*<0.05, Mann Whitney U test) (Supplementary Table 3). This suggests that moderate AD patients have a richer gut bacterial profile, while patients without AD have a less diverse profile. Moreover, MRSA gut colonization was not associated with any differences in the richness and diversity estimates in the AD groups.

**Fig. 3:**
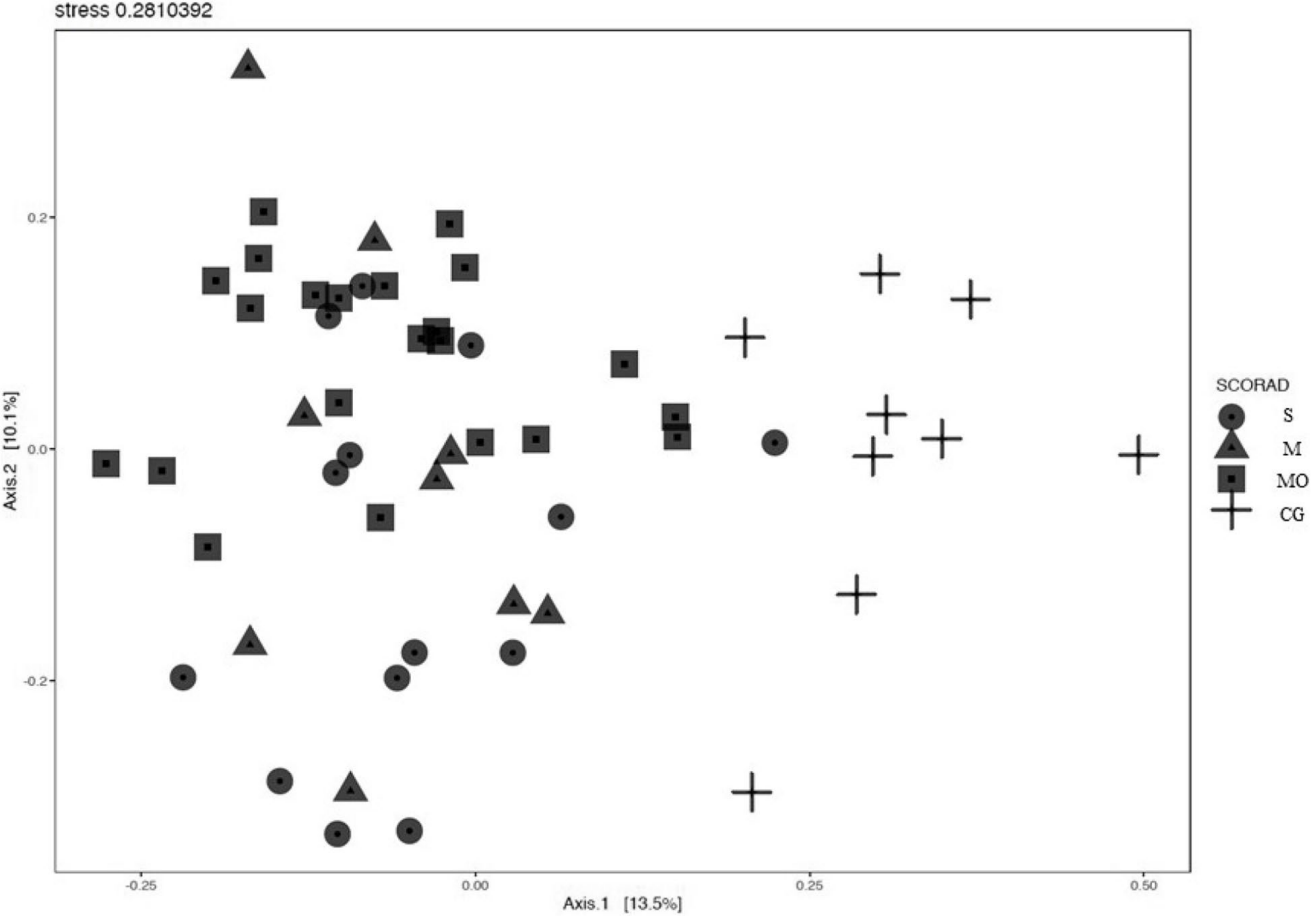
Score plot of principal component analysis based on DGGE profiles of the gut bacterial community in AD patients and control group. AD – atopic dermatitis; SCORAD: S – severe, M – mild, MO – moderate, CG – control group

To visually represent differences in the gut bacterial profiles of AD patients and the control group, we performed principal component analysis (PCA) based on PCR-DGGE banding profiles (Fig. 3). The score plot shows that most AD patient samples were located on the left side of the plot, while most of the control group samples were on the right. In addition, samples from moderate AD patients were closely clustered, indicating a more similar bacterial community among them, as compared to mild and severe AD patients. These findings suggest that the bacterial profile of AD patients exhibits greater dissimilarity from the control group than it does among varying severities of AD.

## Discussion

*Staphylococcus aureus* is a common pathogen that can be isolated from the nares and skin of almost all AD patients and is known to exacerbate the disease [32]. Recent studies have reported that this pathogen may also colonize the intestines of atopic dermatitis patients, but its role in the disease is still unclear [33]. In this study, we aimed to characterize *Staphylococcus* spp. isolates from the nares and feces of AD pediatric patients and compare the gut bacterial community with non-AD children. Our findings revealed high levels of *S. aureus* colonization, including MRSA isolates, in both niches among AD patients, and qualitative differences in gut bacterial profiles were observed between the groups. These results highlight the importance of monitoring and microbiological surveillance of different colonization sites in patients with AD.

AD patients are about five times more likely to carry the pathogen in the nose compared to healthy controls [34–36], and several studies have already reported *S. aureus* nasal prevalence up to 80% among those patients [37–39]. Likewise, we found a high abundance of *S. aureus* (81.8%) in the nares of the AD group, highlighting its unique presence in 45.5% of them. In Brazil, high rates of *S. aureus* colonization were also previously reported [15, 39, 40]. These findings stress that an impaired skin barrier and the presence of a virulent bacteria like *S. aureus* play a vital role in AD pathogenesis and are correlated with the extent of skin lesions, as well as being a potential source of recolonization [41]. Hence, nares are confirmed as an important reservoir for *S. aureus* in AD patients, and it would be necessary to include this site in anti-staphylococcal therapeutic strategies during AD treatment.

The role of the intestinal colonization by *S. aureus* in AD remains uncertain and controversial. In this study, we found a higher presence of *S. aureus* in the feces of AD patients (70.9%) than in non-AD children (33.3%). Melli et al., (2020) [42] in a study performed in São Paulo, Brazil with 81 children, 21 diagnosed with AD and 58 health individuals, found high rates of *S. aureus* gut colonization in AD group (52.2%), while high values (55.2%) were also reported in the control group. However, in healthy individuals, the pathogen’s rate in the gut has ranged from 3% to 13.8% [12, 43]. In AD patients, similarly to the skin, higher rates of *S. aureus* gut colonization are commonly observed, ranging from 50 to 60% [44, 45]. The gastrointestinal tract has been reported as a potential reservoir for *S. aureus* and it could be an important risk factor for AD patients as it can lead to increased rates of infection, host-to-host transmission, and environmental contamination [46–49]. Furthermore, nasal carriage has been associated with increased *S. aureus* intestinal colonization, suggesting a close relationship between the niches [50]. For instance, Squier et al., (2002) [51] showed that pediatric patients carrying *S. aureus* in both the gut and nares were more likely to develop staphylococcal infections than nasal carriers alone (40% vs. 18%, *p*-value < 0.001). Similarly, in children with cancer, MRSA nasal and intestinal colonization were associated with a significantly higher risk of infection compared to just one site [52]. Thus, since most *S. aureus* infections are generally preceded by commensal colonization and it plays a crucial part in AD onset and worsening, intestinal colonization should also take into consideration in disease therapeutic challenges/strategies.

Although some studies suggest that *S. aureus* play an important role in the microbiota of the intestinal tract, stimulating immune system maturation in early childhood [53, 54] it has also been associated with dysbiosis in the intestinal tract, especially in patients with AD compared to healthy individuals, indicating a potential role in the development, and worsening of the disease [55, 56]. Our results showed dissimilarity between the AD and control groups in terms of gut bacterial profiles showing distinct differences, suggesting a possible dysbiosis in AD patients. We also observed a richer profile of gut bacterial in moderate AD patients. The longer interval between AD flares, less use of antimicrobials due to fewer bacterial infections, and absence of immunosuppressant drugs in moderate AD patients compared to severe AD patients could explain the richer profile observed in that group. Penders and colleagues (2006) [57] compared the gut microbiota of AD infants who developed eczema within the first year of life and healthy children by DGGE and they found similar band richness in both groups. However, the authors also described an association between pathogenic bacteria, such as genus *Escherichia coli,* in the gut and a higher tendency towards atopy in one-month old children. Thus, our results may emphasize the relationship between the bacterial intestinal community and AD, although more studies are needed. However, the influence of gut microbiota on the disease does not appear to be limited to its composition but rather is a multifactorial relationship that includes composition, immunological factors, and the use of antimicrobials and immunosuppressant drugs.

The methicillin resistance may allow *S. aureus* to persist in the nares, skin, and gastrointestinal tract of patients with AD [58]. In this study, MRSA isolates were found in 43.6% of AD patients in at least one of the clinical sites surveyed. Worldwide, rates of MRSA colonization in patients with AD have ranged from 16% to 57.4% [15, 17, 37, 39, 59]. For example, Ali et al. (2019) [59] found MRSA isolates in 57.4% of lesioned skin and nares samples from AD patients attending clinics in Egypt. However, the prevalence of MRSA isolates can vary between countries and even within regions of the same country. Cavalcante and colleagues (2015) [15] demonstrated a lower prevalence of MRSA isolates (23%) recovered from nares and lesioned skin of AD patients attending a hospital in Southeast Brazil, while Petry and coworkers [40] did not find any MRSA isolates colonizing AD patients in their study conducted in South region of Brazil. Abad and coworkers [39] found that 27.4% of pediatric AD patients attending a public hospital in Brazil were colonized by MRSA isolates in the nares, with higher incidence in moderate and severe cases. As patients with AD are more susceptible to *S. aureus* infections and frequently attend healthcare settings, they may also use topical and systemic antibiotics, which can increase MRSA colonization and antimicrobial resistance. It is worth mentioning that MRSA isolates from clone USA800/ST5 are very frequent in hospitals in Rio de Janeiro [60, 61] and stood out in our study, which could explain this greater resistance found. High rates of MRSA isolates represent a therapeutic challenge, as β-lactam drugs are the first choice for treating staphylococcal skin infections in AD patients and more aggressive antibiotic therapy may be required.

Although *S. aureus* is undoubtedly the most relevant pathogen in AD, the role of CoNS remains unclear. In our study, these isolates were identified in 72.7% of patients, 29 (52.7%) of them coming from the nares. *S. epidermidis* was the most prevalent species detected (56.4%). Likewise, Ndhlovu et al., (2022) [62] observed similar rates (56.7%) of CoNS in anterior nares from AD children in South Africa. Indeed, some studies have suggested a potential pathogenic role for CoNS in AD [32]. Byrd et al., 2017 [63] found higher abundance of *S. epidermidis* during AD flares when compared to pos-flares. In fact, *S. epidermidis* strains can contribute to AD worsening through the production of the cysteine protease EcpA, which promotes epidermal damage and inflammation [64]. Moreover, we detected high rates of nasal and fecal MRSE isolates. In fact, our group had already reported a high incidence (60%) of MRCoNS from the nares, lesional, and non-lesional skin of AD patients attended in the same dermatological service as the present study, and most of these isolates were classified as MRSE [17]. Similarly, Byrd et al., (2017) [63] also reported a predominance of MRSE colonizing AD patients. Therefore, even though CoNS isolates are known as commensal microorganisms, many of them are related to antimicrobial resistance and are considered reservoirs of transferable resistance/virulence genes to *S. aureus*, such as SCC*mec* and ACME elements [65–67].

The *S. aureus* clonal lineages are present in both hospital and community environments, and the most common clones in Brazilian AD patients are those that are well-established in the community, such as USA800/ST5/SCC*mec*IV and USA1100/ST30/SCC*mec*IV [15, 17, 68]. In this study, we identified at least five clonal lineages and 13 *spa* types, and as observed in previous studies [17, 39, 68], most isolates belonged to USA800/SCC*mec*IV (47.6%) and USA1100/SCC*mec*IV (21.4%) and the *spa* types most found were t002 and t318, respectively. These major community lineages also colonize asymptomatically healthy individuals as causing mild to life-threatening infections [17, 61, 68]. Interestingly, PVL was mainly detected in these lineages, which has also been reported by Cavalcante et al., in Brazil [15, 68].

Expression of PVL may aggravate AD through various mechanisms [69]. In this study, 13 *S. aureus* isolates from 10 (18.2%) AD patients were positive for *pvl* genes. Furthermore, these genes were more found among severe AD patients (*p*-value < 0.05). Therefore, these findings indicate that, despite the great *S. aureus* clonal diversity among AD patients, there is a predominance of virulent community-related lineages that may increase colonization rates and lead to more severe forms of AD. Moreover, we found that 61.9% (13/21) of patients had genetically related isolates in their nostrils and feces. This is consistent with several previous studies that have shown patients to be colonized by genotypically identical isolates in their nares and lesioned skin [15, 17, 69–71]. These results suggest that colonized niches may serve as a focus for a new colonization episode or to maintain the pathogen in the environment.

While this study is the first characterization of *Staphylococcus* from feces of children with AD in Brazil, it is important to acknowledge some limitations. First, the sample size was relatively small. Secondly, moderate AD patients were more present in the study population. This may be because the Dermatology Service observed in the present study is a reference center of AD treatment in Rio de Janeiro, and moderate AD patients may be more likely to seek medical attention.

## Conclusion

AD pediatric patients showed high rates of colonization by *S. aureus* and methicillin-resistant staphylococci isolates. In addition, gut bacterial profiles of AD patients grouped differently from those of the control group, highlighting the importance of monitoring colonization by *S. aureus* and the gut microbiome composition in these patients and their role in disease aggravation.

### Supplementary Information

**Additional file 1.** 

**Additional file 2: Supplementary Table 1.** General characteristics associated to 55 patients with atopic dermatitis and nine control individuals colonized by *Staphylococcus* spp.

**Additional file 3: Supplementary Table 2.** Antimicrobial susceptibility of 84 *Staphylococcus aureus* isolates recovered from atopic dermatitis patients.

**Additional file 4:Supplementary Table 3.** Chao and Shanon indexes associated with 45 AD pediatric patients and nine evaluated controls.

## Data Availability

The data sets generated and analyzed during the current study, such as PFGE/DGGE are not public available as there is no public database to deposit PFGE/DGGE results. Although no new *spa* types were found in the present study, data on *spa* type DNA sequences are available in the Supplementary file.

## Abbreviations

AD: Atopic dermatitis
*spa*: Gene coding protein A
MRSA: Methicillin-resistant *Staphylococcus aureus*
MSSA: Methicillin-susceptible *S. aureus*
SCC*mec*: Staphylococcal cassette chromosome *mec*
SCORAD: Scoring atopic dermatitis
TE: Tris-EDTA
HCL: Hydrochloric acid
BD: Becton and Dickson
USA: United State of America
MALDI-TOF/MS: Matrix assisted laser desorption mass spectrometry
CLSI: Clinical and Laboratory Standards Institute
TMP/STX: Trimethoprim-sulfamethoxazole
UK: United Kingdom
MIC: Minimum inhibitory concentration
MDR: Multidrug resistant
ATCC: American type culture collection
DNA: deoxyribonucleic acid
MRSE: Methicillin-resistant *Staphylococcus epidermidis*
MSSE: Methicillin-susceptible *S. epidermidis*
PVL: Panton-Valentine leucocidin
PFGE: Pulsed-field gel electrophoresis
UPGMA: Unweighted pair-group method arithmetic
PCR: Polymerase chain reaction
DGGE: Denaturing Gradient Gel Electrophoresis
rRNA: ribosomal ribonucleic acid
TAE: Tris-Acetate-EDTA
PCA: Principal component analysis
CoNS: Coagulase-negative staphylococci
CA-MRSA: Community-acquired MRSA
SD: Standard deviation
EcpA: Cysteine protease
MR-CoNS: Methicillin-resistant CoNS
ACME: Arginine catabolic mobile element
