# Supplementary material for: Atopic dermatitis pediatric patients show high rates of nasal and intestinal colonization by methicillin-resistant Staphylococcus aureus and coagulase-negative staphylococci
Source: BMC Microbiol. 2024 Jan 29;24:42. doi: 10.1186/s12866-023-03165-5 (PMC10823624; doi:10.1186/s12866-023-03165-5)
Supplement: Supplementary file 1 — Additional file 1. [file 12866_2023_3165_MOESM1_ESM.docx]

**Isolate n° 184n - t065 spa type** TTTAAAAGAGGACCTTCGGTGAGCAAAGRAAKTTTAGCAGAAGCTAAAAAGCTAAACGATGCTCAAGCACCAAAAGAGGAAGACGGCAACAAACCTGGTAAAGAAGACAACAAAAAACCTGGCAAAGAAGACGGCAACAAACCTGGTAAAGAAGACAACAAAAAACCTGGTAAAGAAGACAACAACAAACCTGGTAAAGAAGACGGCAACAAGCCTGGTAAAGAAGACAACAAAAAACCTGGTAAAGAAGACGGCAACAAACCTGGTAAAGAAGACAACAAAAAACCTGGTAAAGAAGACGGCAACGGAGTACATGTCGTTAAACCTGGTGATACAGTAAATGACATYGCAAAAGCAAACGGCACTAMCTGCTGA

**Isolate n° 184f - t002 spa type**

AATTTWAARGGACGGTCCTTCGGTGAGCAAAGRCCACARWWTTAGCAGAAGCTAAAAAGCTAAACGATGCTCAAGCACCAAAAGAGGAAGACAACAAAAAACCTGGTAAAGAAGACGGCAACAAACCTGGCAAAGAAGACGGCAACAAGCCTGGTAAAGAAGACAACAAAAAACCTGGTAAAGAAGACGGCAACAAGCCTGGTAAAGAAGACAACAACAAACCTGGCAAAGAAGACGGCAACAAGCCTGGTAAAGAAGACAACAACAAGCCTGGTAAAGAAGACGGCAACAAGCCTGGTAAAGAAGACGGCAACAAACCTGGTAAAGAAGACGGCAACGGAGTACATGTCGTTAAACCTGGTGATACAGTAAATGACATTRSARAARCAARCGCCYTTMMYKSCTGA

**Isolate n° 194n - t318 spa type**

TTTTAAAGATTATCGTTGATTGATCAAWGAAMTTWGAGCTGWTGGTAGAAAGTTAAAYGATGMTCGATGCACCAAGCAGCTGACAACAAATTCAACAAAGACCAAGAACATGATTTTTATTTAATTTTAATTTTACTTATATTAAATGTAGMWSAASGTMAMGGYWTCGTCCAAAGCCTRASCYTTAAAAGAGGATCTTCGGTGAGCAAAGRAATTTTAGCAGAAGCTAAAAAGCTAAACGATGCTCAAGCACCAAAAGAGGAAGACAACAACAAGCCTGGCAAAGAAGACAACAACAAGCCTGGTAAAGAAGACGGCAACAAACCTGGTAAAGAAGACGGCAACAAACCTGGTAAAGAAGACAACAAAAAACCTGGCAAAGAAGACGGCAACAAACCTGGTAAAGAAGACAACAAAAAACCTGGCAAAGAAGATGGCAACAAACCTGGTAAAGAAGACGGCAACAAGCCTGGTAAAGAAGATGGCAACAAGCCTGGTAAAGAAGACGGCAACGGAGTACATGTCGTTAAACCTGGTGATACAGWAAATGACWTTGSMAAMRCAAACGGCACTACTGCTGAGA

**Isolate n° 194f - t318 spa type**

TTAAAAGACGGATCCTTCGGTGAGCAAAGAAATTTTRGCAGGARGCTAAAAAGCTAAACGATGCTCAAGCACCAAAAGAGGAAGACAACAACAAGCCTGGCAAAGAAGACAACAACAAGCCTGGTAAAGAAGACGGCAACAAACCTGGTAAAGAAGACGGCAACAAACCTGGTAAAGAAGACAACAAAAAACCTGGCAAAGAAGACGGCAACAAACCTGGTAAAGAAGACAACAAAAAACCTGGCAAAGAAGATGGCAACAAACCTGGTAAAGAAGACGGCAACAAGCCTGGTAAAGAAGATGGCAACAAGCCTGGTAAAGAAGACGGCAACGGAGTACATGTCGTTAAACCTGGTGATACAGTAAATGACATWGCAAAAGCAAACGGACYTMCYGCTGA

**Isolate n° 195f - t002 spa type**

TTWAAARRTTGACTTCGGTGAGCAAAGARRWTTTAGCAGAAGCTAAAAAGCTAAACGATGCTCAAGCACCAAAAGAGGAAGACAACAAAAAACCTGGTAAAGAAGACGGCAACAAACCTGGCAAAGAAGACGGCAACAAGCCTGGTAAAGAAGACAACAAAAAACCTGGTAAAGAAGACGGCAACAAGCCTGGTAAAGAAGACAACAACAAACCTGGCAAAGAAGACGGCAACAAGCCTGGTAAAGAAGACAACAACAAGCCTGGTAAAGAAGACGGCAACAAGCCTGGTAAAGAAGACGGCAACAAACCTGGTAAAGAAGACGGCAACGGAGTACATGTCGTTAAACCTGGTGATACAGTAAATGACATKSCAAAAGCAAACGGCCTTWMCTGCTGAACTCACCGAAAAT

Isolate n° 198f1 - t037 spa type

CCTTATTTAAARGACGGACCTTCGGTGAGCAAAGAAATTTWRGCAGRAGGCTAAAAAGCTAAACGATGCTCAAGCACCAAAAGAGGAAGACAACAACAAGCCTGGCAAAGAAGACAACAACAAGCCTGGTAAAGAAGACGGCAACAAACCTGGTAAAGAAGACAACAAAAAACCTGGCAAAGAAGATGGCAACAAACCTGGTAAAGAAGACGGCAACAAGCCTGGTAAAGAAGATGGCAACAAGCCTGGTAAAGAAGACGGCAACGGAGTACATGTCGTTAAACCTGGTGATACAGTAAATGACATTGCAAAAGCAAACGGACTWACTGCTGA

**Isolate n° 199n1 - t6726 spa type**

TTAAAAGAYTATTCCTTCGGTGAGCAAAGAAATTTTRGCAGWAGCTAAAAAGCTAAAYGATGCWCAAGCACCAAAAGYTGACAACAAATTMAACAAAGAACAACAAAATRCWTTATATGAAATTTTACATTTACCTAAYTTAAATGAAGAACAACGTAAYGGCTTCATCCTTATCCTAAGTTTAAAGATGRTCTTCGGTGAGCAAAGAAWTTTTAGSMRRAGCTAAAAAGCTAAACGATGCTCAAGCACCAAAAGAGGAAGACAACAACAAGCCTGGCAAAGAAGACAACAACAAGCCTGGTAAAGAAGACGGCAACAAACCTGGTAAAGAAGACGGCAACAAACCTGGTAAAGAAGACGGCAACAAACCTGGTAAAGAAGACAACAAAAAACCTGGCAAAGAAGACGGCAACAAACCTGGTAAAGAAGACAACAAAAAACCTGGCAAAGAAGATGGCAACAAACCTGGTAAAGAAGACGGCAACAAGCCCTGGTAAAGAAGATGGCAACAAGCCTGGTAAAGAAGACGGCAACGGAGTACATGTCGTTAAACCTGGTGATACAGTAAATGACWKWGCRRWAKCRRACGGACTAMCTGCTGAAWCTCTACTRAAAAAGGCTWTGGAWRAAKCCGTTACATTGTTCTTCAGTTAAKTTAGGTATATGTAAAATTTCRTAGAAAGCATTTTGTTGTTCTTTGTTGAATTTGWWGTCAKCTSTYGSTGCWKRWGCATYATTTARCTTTTTAGCTTCTGCTAAAATTTCTTTGCTCACCGAAGATCGTCTTTAAAAA

**Isolate n° 199f1 - t1130 spa type**

TTTAARRAGGWCCTTCGGTGAGCAAAGAAATTTTAGSWGAMGCTAAAAAGCTAAACGATGCTCAAGCACCAAAAGAGGAAGACAACAACAAGCCTGGCAAAGAAGACAACAACAAGCCTGGTAAAGAAGACGGCAACAAACCTGGTAAAGAAGACGGCAACAAACCTGGTAAAGAAGACGGCAACAAACCTGGTAAAGAAGACAACAAAAAACCTGGCAAAGAAGACGGCAACAAACCTGGTAAAGAAGACAACAAAAAACCTGGCAAAGAAGATGGCAACAAACCTGGTAAAGAAGACGGCAACAAGCCTGGTAAAGAAGATGGCAACAAGCCTGGTAAAGAAGACGGCAACGGAGTACATGTCGTTAAACCTGGTGATACAGTAAATRMCATTGCAAAAGCAAACG

**Isolate n° 200n - t1451 spa type**

GATTTTTTAAAGACGGMCCTTCGGTGAGCRARGAAATTTTAGSCARGAAGCTAAAAAGCTAAACGATGCTCAAGCACCAAATAGAGGAAGACAACAACAAGCCTGGTAAAGAAGACGGCAACAAACCTGGTAAAGAAGACAACAAAAAACCTGGCAAAGAAGATGGCAACAAACCTGGTAAAGAAGACAACAAAAAACCTGGTAAAGAAGATGGCAACAAACCTGGTAAAGAAGACGGCAACGGAATACATGTCGTTAAACCTGGTGATACAGTAAATGACMWTGCAAAAGCAAACGGCACTTACTGCTGA

**Isolate n° 200f - t037 spa type**

TCCGTTTAAAAGACGGACCTTCGGTGAGCAAAGRAATTTTAGCAGAAGCTAAAAAGCTAAACGATGCTCAAGCACCAAAAGAGGAAGACAACAACAAGCCTGGCAAAGAAGACAACAACAAGCCTGGTAAAGAAGACGGCAACAAACCTGGTAAAGAAGACAACAAAAAACCTGGCAAAGAAGATGGCAACAAACCTGGTAAAGAAGACGGCAACAAGCCTGGTAAAGAAGATGGCAACAAGCCTGGTAAAGAAGACGGCAACGGAGTACATGTCGTTAAACCTGGTGATACAGTAAATRMCATTGCAAAAGCAAACGGACTWACTGCTGAACTC

**Isolate n° 201n1 - t5189 spa type**

TTAAAAGATGACCTTCGGTGAGCAAAGARATKTTAGSARARGYWAAAAAGCTAAACGATGCTCAAGCACCAAAAGAGGAAGACAACAACAAACCTGGTAAAGAAGACGGCAACAAACCTGGCAAAGAAGACAACAACAAGCCTGGTAAAGAAGACAACAAAAAACCTGGTAAAGAAGACAACAACAAGCCTGGTAAAGAAGACAACAACAAGCCTGGTAAAGAAGACAACAACAAGCCTGGTAAAGAAGACGGCAACAAACCTGGCAAAGAAGACAACAACAAGCCTGGTAAAGAAGACGGCAACAAACCTGGCAAAGAAGACGGCAACGGAGTACATGTCGTTAAACCTGGTGATACAGTAAATRACATTGCAAAAGCAAACGGCACTACTGCTGA

**Isolate n° 201f - t5189 spa type**

TTWAAAGACGAACCTTCGGTGAGCAAAGAAATTTKRGCAAGGARGCTAAAAAGCTAAACGATGCTCAAGCACCAAAAGAGGAAGACAACAACAAACCTGGTAAAGAAGACGGCAACAAACCTGGCAAAGAAGACAACAACAAGCCTGGTAAAGAAGACAACAAAAAACCTGGTAAAGAAGACAACAACAAGCCTGGTAAAGAAGACAACAACAAGCCTGGTAAAGAAGACAACAACAAGCCTGGTAAAGAAGACGGCAACAAACCTGGCAAAGAAGACAACAACAAGCCTGGTAAAGAAGACGGCAACAAACCTGGCAAAGAAGACGGCAACGGAGTACATGTCGTTAAACCTGGTGATACAGTAAAWGACATTGCAAAAGCAAACGGCACTAMCCTGCTGA

**Isolate n° 202n - t5189 spa type**

TTAAAGATGTCTTCGGTGAGCAAAGAAATTTTRGCAGRAGGCTAAAAAGCTAAACGATGCTCAAGCACCAAAAGAGGAAGACAACAACAAACCTGGTAAAGAAGACGGCAACAAACCTGGCAAAGAAGACAACAACAAGCCTGGTAAAGAAGACAACAAAAAACCTGGTAAAGAAGACAACAACAAGCCTGGTAAAGAAGACAACAACAAGCCTGGTAAAGAAGACAACAACAAGCCTGGTAAAGAAGACGGCAACAAACCTGGCAAAGAAGACAACAACAAGCCTGGTAAAGAAGACGGCAACAAACCTGGCAAAGAAGACGGCAACGGAGTACATGTCGTTAAACCTGGTGATACAGTAAATRAMATTGCAAAAGCAAACGGACTWMCYKGCTGA

**Isolate n° 202f1 - t180 spa type**

CCTTTAAAAGAGGACCTTCGGTGAGCAAARAAWTTTTAGCAGAAGCTAAAAAGCTAAACGATGCTCAAGCACCAAAAGAGGAAGACGGCAACAAACCTGGTAAAGAAGACAACAAAAAACCTGGCAAAGAAGACGGCAACAAACCTGGTAAAGAAGACAACAAAAAACCTGGTAAAGAAGACAACAAAAAACCTGGTAAAGAAGACAACAAAAAACCTGGTAAAGAAGACGGCAACAAGCCTGGTAAAGAAGACAACAAAAAACCTGGTAAAGAAGACGGCAACAAACCTGGTAAAGAAGACAACAAAAAACCTGGTAAAGAAGACGGCAACGGAGTACATGTCGTTAAACCTGGTGATACAGTAAATGMYRWKGYAARAGCAAACGGACYTWACYTGCTGA

**Isolate n° 203n -t067 spa type**

TTWAAAAGAGGGTCTTCGGTGAGCAAAGAAATTTKRGCAGRAGGCTAAAAAGCTAAACGATGCTCAAGCACCAAAAGAGGAAGACAACAAAAAACCTGGTAAAGAAGACGGCAACAAACCTGGCAAAGAAGACGGCAACAAGCCTGGTAAAGAAGACAACAAAAAACCTGGTAAAGAAGACGGCAACAAGCCTGGTAAAGAAGACAACAACAAACCTGGCAAAGAAGACGGCAACAAGCCTGGTAAAGAAGACAACAACAAGCCTGGTAAAGAAGACGGCAACAAGCCTGGTAAAGAAGACGGCAACAAACCCTGGTAAAGAAGACGGCAACGGAGTACATGTCGTTAAACCTGGTGATACAGTAAATGACATYGCAAAAGCAAACGACTAACTGYTGAAC

**Isolate n° 204n - t002 spa type**

TTAARGACGATCCTTCGGTGAGCARAGAAWTTTTAAGCAGAAGCTAAAAAGCTAAACGATGCTCAAGCACCAAATAGAGGAAGACAACAAAAAACCTGGTAAAGAAGACGGCAACAAACCTGGCAAAGAAGACGGCAACAAGCCTGGTAAAGAAGACAACAAAAAACCTGGTAAAGAAGACGGCAACAAGCCTGGTAAAGAAGACAACAACAAACCTGGCAAAGAAGACGGCAACAAGCCTGGTAAAGAAGACAACAACAAGCCTGGTAAAGAAGACGGCAACAAGCCTGGTAAAGAAGACGGCAACAAACCTGGTAAAGAAGACGGCAACGGAGTACATGTCGTTAAACCTGGTGATACAGTTAAATGACAMTTGCAAAAGCAAACGGCACTACTGCTGACC

**Isolate n° 212n - t002 spa type**

CTTTTTAAAGAYGATCSTTSGGTGAGCAAAGAAATTTTAGCAGAAGSTAAAAAGYTAAAYGATGCWCAAGCACCAAAAGCTGACAACAAATTCAACAAAGAMCAACAAAATGMTTTYTATGAAATTTTACATTTACMTWWCTTAAMTGAAGAACAASGTAAMGGCTTCATCCAAAGCCTAGCCTTAAAGACGATCCTTCGGTGAGCAAAGAAATTTTWGCAGAAGCTWAAAAGCTAAACGATGCTCAAGCACCAAAAGAGGAAGACAACAAAAAACCTGGTAAAGAAGACGGCAACAAACCTGGCAAAGAAGACGGCAACAAGCCTGGTAAAGAAGACAACAAAAAACCTGGTAAAGAAGACGGCAACAAGCCTGGTAAAGAAGACAACAACAAACCTGGCAAAGAAGACGGCAACAAGCCTGGTAAAGAAGACAACAACAAGCCTGGTAAAGAAGACGGCAACAAGCCTGGTAAAGAAGACGGCAACAAACCTGGTAAAGAAGACGGCAACGGAGTACATGTCGTTAAACCTGGTGATACAGTAAATGACMWKGCAAAAGCAAACGGCACTACTGCTGA

**Isolate n° 212f - t002 spa type**

CTTAAAGACGTCCTTCGGTGAGCAAAGAAATTTTAGSAGARGYTAAAAAGCTAAACGATGCTCAAGCACCAAAAGAGGAAGACAACAAAAAACCTGGTAAAGAAGACGGCAACAAACCTGGCAAAGAAGACGGCAACAAGCCTGGTAAAGAAGACAACAAAAAACCTGGTAAAGAAGACGGCAACAAGCCTGGTAAAGAAGACAACAACAAACCTGGCAAAGAAGACGGCAACAAGCCTGGTAAAGAAGACAACAACAAGCCTGGTAAAGAAGACGGCAACAAGCCTGGTAAAGAAGACGGCAACAAACCTGGTAAAGAAGACGGCAACGGAGTACATGTCGTTAAACCTGGTGATACAGTAAATRMCATTGCAAAAGCAAACGGCACTTACCTGCTGA

**Isolate n° 214f - t002 spa type**

CTTTAAAAGATGGACCTTCGGTGAGCAARGAAATTTTARCMRRAGCTAAAAAGCTAAACGATGCTCAAGCACCAAAAGAGGAAGACAACAAAAAACCTGGTAAAGAAGACGGCAACAAACCTGGCAAAGAAGACGGCAACAAGCCTGGTAAAGAAGACAACAAAAAACCTGGTAAAGAAGACGGCAACAAGCCTGGTAAAGAAGACAACAACAAACCTGGCAAAGAAGACGGCAACAAGCCTGGTAAAGAAGACAACAACAAGCCTGGTAAAGAAGACGGCAACAAGCCTGGTAAAGAAGACGGCAACAAACCTGGTAAAGAAGACGGCAACGGAGTACATGTCGTTAAACCTGGTGATACAGTAAATGACMWTGCMMAAGCAAACGGACTTWCCYKSCTGA

**Isolate n° 216n - t002 spa type**

ATTWWWGKACGATCCTTCGGTGAGCAAWGAAATTTTRGCAGRAGGCTAAAAAGCTAAACGATGCTCAAGCACCAAAAGAGGAAGACAACAAAAAACCTGGTAAAGAAGACGGCAACAAACCTGGCAAAGAAGACGGCAACAAGCCTGGTAAAGAAGACAACAAAAAACCTGGTAAAGAAGACGGCAACAAGCCTGGTAAAGAAGACAACAACAAACCTGGCAAAGAAGACGGCAACAAGCCTGGTAAAGAAGACAACAACAAGCCTGGTAAAGAAGACGGCAACAAGCCTGGTAAAGAAGACGGCAACAAACCTGGTAAAGAAGACGGCAACGGAGTACATGTCGTTAAACCTGGTGATACCAGTAAATGMCMTTGCAAAAGCAAACGGACYWMCYTGCTGA

**Isolate n° 218f - t1154 spa type**

TTTTTTAAAGGACGACCTTCGGTGAGCAAAGAAATTTTAGCAGAAGCTAAAAAGCTAAACGATGCTCAAGCACCAAAAGAGGAAGACAACAAAAAACCTGGTAAAGAAGACAACAACAAACCTGGCAAAGAAGACGGCAACAAGCCTGGTAAAGAAGACAACAACAAGCCTGGTAAAGAAGACGGCAACAAGCCTGGTAAAGAAGACGGCAACAAACCTGGTAAAGAAGACGGCAACGGAGTACATGTCGTTAAACCTGGTGATACAGTAAAKGACCAG

**Isolate n° 221n1 - t5693 spa type**

TTAAAGACGATCCTTCGGTGAGCATTAAAATTKTTGCTGAAGYTAAAAAAGTAATTGATGCTCAAGCTCCAAAAGTGGAAGAYAACAACAARCCARTTAAAGAMGATCCTTMGGTGAGCAAAGAAATTTKRGCAGGAAGSYWAAAAAGCTAAACGATGCTCAAGCMCCAAAAGAGGAAGACAACAACAAACCTGGTAAAGAAGACGGCAACAAACCTGGCAAAGAAGACAACAACAAGCCTGGTAAAGAAGACAACAACAAGCCTGGCAAAGAAGACGGCAACAAAGCCTGGTAAAGAAGACAACAAAAAACCTGGTAAAGAAGATGGCAACGGAGTACATGTCGTTAAACCTGGTGATACAGTAAATRMCWTWGSWWARACAAACGGCACTACTGCT

**Isolate n° 221f - t189 spa type**

GAATTTTGCAGAGCTAAAAAGCTAAACGATGCTCAAGCACCAAAAGAGGAAGACAACAACAAACCTGGTAAAGAAGACGGCAACAAACCTGGCAAAGAAGACAACAACAAGCCTGGTAAAGAAGACAACAACAAGCCTGGCAAAGAAGACGGCAACAAGCCTGGTAAAGAAGACAACAAAAAACCTGGTAAAGAAGATGGCAACGGAGTACATGTCGTTAAACCTGGTGATACAGTAAATGACATTGCAAAAGCAAACGGACTTMCTGCTGA

**Isolate n° 224f - t189 spa type**

GAATTTTGCAGAGCTAAAAAGCTAAACGATGCTCAAGCACCAAAAGAGGAAGACAACAACAAACCTGGTAAAGAAGACGGCAACAAACCTGGCAAAGAAGACAACAACAAGCCTGGTAAAGAAGACAACAACAAGCCTGGCAAAGAAGACGGCAACAAGCCTGGTAAAGAAGACAACAAAAAACCTGGTAAAGAAGATGGCAACGGAGTACATGTCGTTAAACCTGGTGATACAGTAAATGACATTGCAAAAGCAAACGGACTTMCTGCTGA

**Isolate n° 225n - t318 spa type**

ACTTAAAGAGGTCCTTCGGTGAGCRAARAWWTTTTRGCAAGGARGCTAAAAAGGCTAAACGATGCTCAAGCACCAAAAGAGGAAGACAACAACAAGCCTGGCAAAGAAGACAACAACAAGCCTGGTAAAGAAGACGGCAACAAACCTGGTAAAGAAGACGGCAACAAACCTGGTAAAGAAGACAACAAAAAACCTGGCAAAGAAGACGGCAACAAACCTGGTAAAGAAGACAACAAAAAACCTGGCAAAGAAGATGGCAACAAACCTGGTAAAGAAGACGGCAACAAGCCTGGTAAAGAAGATGGCAACAAGCCTGGTAAAGAAGACGGCAACGGAGTACATGTCGTTAAACCTGGTGATACAGTAAATRMCATTGCAAAAGCAAACGGACTWMCTGCTGA

**Isolate n° 228f1 - t002 spa type**

TTAAWGACGATCCTTCGGTGAGSAAARAWWTTTTAGCAGRAGGCTAAAAAGCTAAACGATGCTCAAGCACCAAAAGAGGAAGACAACAAAAAACCTGGTAAAGAAGACGGCAACAAACCTGGCAAAGAAGACGGCAACAAGCCTGGTAAAGAAGACAACAAAAAACCTGGTAAAGAAGACGGCAACAAGCCTGGTAAAGAAGACAACAACAAACCTGGCAAAGAAGACGGCAACAAGCCTGGTAAAGAAGACAACAACAAGCCTGGTAAAGAAGACGGCAACAAGCCTGGTAAAGAAGACGGCAACAAACCTGGTAAAGAAGACGGCAACGGAGTACATGTCGTTAAACCTGGTGATACAGTAAATRACATTGCAAAAGCAAACGGCACTACTGCTGA

**Isolate n° 234f1 - t002 spa type**

TTAAAAGACGGACCTTCGGTGAGCAAAGAAATTKKMGCRGAAGCTAAAAAGCTAAACGATGCTCAAGCACCAAAAGAGGAAGACAACAAAAAACCTGGTAAAGAAGACGGCAACAAACCTGGCAAAGAAGACGGCAACAAGCCTGGTAAAGAAGACAACAAAAAACCTGGTAAAGAAGACGGCAACAAGCCTGGTAAAGAAGACAACAACAAACCTGGCAAAGAAGACGGCAACAAGCCTGGTAAAGAAGACAACAACAAGCCTGGTAAAGAAGACGGCAACAAGCCTGGTAAAGAAGACGGCAACAAACCTGGTAAAGAAGACGGCAACGGAGTACATGTCGTTAAACCTGGTGATACAGTAAATGACATTGCAAAAGCAAACGGACTWWMYKGCTGA
