# Supplementary material for: Atopic dermatitis pediatric patients show high rates of nasal and intestinal colonization by methicillin-resistant Staphylococcus aureus and coagulase-negative staphylococci
Source: BMC Microbiol. 2024 Jan 29;24:42. doi: 10.1186/s12866-023-03165-5 (PMC10823624; doi:10.1186/s12866-023-03165-5)
Supplement: Supplementary file 2 — Additional file 2: Supplementary Table 1. General characteristics associated to 55 patients with atopic dermatitis and nine control individuals colonized by Staphylococcus spp. [file 12866_2023_3165_MOESM2_ESM.doc]

| **Supplementary Table 1: General characteristics associated to 55 patients with atopic dermatitis and nine control individuals colonized by *Staphylococcus* spp** | | | | | | | | | |
| --- | --- | --- | --- | --- | --- | --- | --- | --- | --- |
| **ATOPIC DERMATITIS PATIENTS** | | | | | | | | | |
| **Patient**  **N°** | **SCORAD** | **Age**  **(years)** | **Gender** | **Clinical source** | **Isolate**  **number** | **Species** | **Methicillin**  **resistance** | **SCC*mec***  **type** | **Antimicrobial resistance profile** |
| **1** | S | 8 | F | Nasal | 181n | *S. aureus* | yes | IV | Fox, Pen |
|  |  |  |  | Feces | 181f | *S. aureus* | no | na | Pen |
| **2** | MO | 10 | M | Nasal | 182n1 | *S. aureus* | no | na | Ery, Pen |
|  |  |  |  | Nasal | 182n2 | *S. epidermidis* | no | na | Ery, Gen, Pen, SXT |
|  |  |  |  | Feces | 182f | *S. epidermidis* | yes | V | Ery, Fox, Pen, SXT, Tet |
| **3** | S | 9 | F | Nasal | 183n | *S. aureus* | no | na | Pen |
|  |  |  |  | Feces | 183f | *S. aureus* | no | na | Ery, Pen |
| **4** | M | 7 | F | Nasal | 184n* | *S. aureus* | no | na | Pen |
|  |  |  |  | Feces | 184f | *S. aureus* | yes | IV | Fox, Pen |
| **5** | MO | 3 | F | Nasal | 185n | *S. epidermidis* | no | na | Pen |
|  |  |  |  | Feces | 185f | *S. haemolyticus* | yes | nd | Cip, Fox, Gen, Pen, SXT |
| **6** | MO | 9 | F | Nasal | 186n | *S. aureus* | yes | IV | Fox, Pen |
|  |  |  |  | Feces | 186f | *S. simulans* | no | na | Pen |
| **7** | MO | 8 | F | Nasal | 187n1 | *S.aureus* | no | na | Pen |
|  |  |  |  | Nasal | 187n2 | *S. epidermidis* | no | na | Ery, Pen |
|  |  |  |  | Feces | 187f1 | *S.aureus* | no | na | Pen |
|  |  |  |  | Feces | 187f2 | *S. epidermidis* | no | na | Ery, Pen |
| **8** | MO | 9 | M | Nasal | 188n | *S. aureus* | no | na | Ery |
|  |  |  |  | Feces | 188f | *S. aureus* | no | na | Cli, Ery, Pen |
| **9** | MO | 9 | M | Nasal | 189n | *S. epidermidis* | yes | nt | Fox, Pen |
|  |  |  |  | Feces | 189f | *S. epidermidis* | yes | V | Cli, Fox, Ery, Pen, SXT |
| **10** | MO | 3 | M | Nasal | 190n1 | *S. aureus* | no | na | Cli, Ery, Gen, Pen |
|  |  |  |  | Nasal | 190n2 | *S. epidermidis* | no | na | Ery, Pen |
|  |  |  |  | Feces | 190f | *S. aureus* | no | na | Cli, Ery, Gen, Pen |
| **11** | M | 9 | F | Nasal | 191n1 | *S. aureus* | no | na | Pen |
|  |  |  |  | Nasal | 191n2 | *S. epidermidis* | yes | nt | Fox, Ery, Pen |
|  |  |  |  | Feces | 191f | *S. warneri* | no | na | Ery, Pen, Tet |
| **12** | M | 9 | M | Nasal | 192n | *S. aureus* | no | na | Cli, Ery, Pen |
|  |  |  |  | Feces | 192f | *S. aureus* | no | na | Ery, Gen, Pen, Tet |
| **13** | M | 9 | F | Nasal | 193n1 | CoNS | no | na | Ery |
|  |  |  |  | Nasal | 193n2 | *S. pasteuri* | no | na | Cli, Ery, Pen |
|  |  |  |  | Feces | 193f1 | *S. aureus* | no | na | Pen |
|  |  |  |  | Feces | 193f2 | *S. epidermidis* | no | na | Ery, Pen, SXT |
| **14** | S | 6 | M | Nasal | 194n* | *S. aureus* | yes | IV | Fox, Pen |
|  |  |  |  | Feces | 194f | *S. aureus* | yes | IV | Fox, Pen |
| **15** | M | 3 | F | Nasal | 195n | *S. aureus* | yes | IV | Fox, Gen, Pen |
|  |  |  |  | Feces | 195f | *S. aureus* | yes | IV | Fox, Gen, Pen |
| **16** | S | 8 | M | Nasal | 196n* | *S. aureus* | yes | IV | Fox, Pen |
|  |  |  |  | Feces | 196f1 | *S. haemolyticus* | no | na | Cli, Ery, Pen, Tet |
|  |  |  |  | Feces | 196f2 | *S. epidermidis* | yes | V | Ery, Fox, Pen, SXT |
| **17** | MO | 5 | M | Nasal | 197n1 | *S. aureus* | no | na | Pen |
|  |  |  |  | Nasal | 197n2 | *S. hominis* | no | na | Pen |
|  |  |  |  | Feces | 197f | *S. haemolyticus* | yes | nd | Ery, Fox,Gen, Pen |
| **18** | S | 6 | F | Nasal | 198n1* | *S. aureus* | yes | IV | Cli, Ery, Fox, Mup, Pen |
|  |  |  |  | Nasal | 198n2 | *S. epidermidis* | yes | nt | Cli, Ery, Fox, Pen, SXT |
|  |  |  |  | Feces | 198f1 | *S. aureus* | yes | III | Cli, Ery, Fox, Pen, Tet |
|  |  |  |  | Feces | 198f2 | *S. epidermidis* | yes | nt | Ery, Fox, Pen, SXT |
| **19** | S | 6 | M | Nasal | 199n1 | *S. aureus* | yes | IV | Fox, Pen |
|  |  |  |  | Nasal | 199n2 | *S. epidermidis* | no | na | Cli, Ery, Pen, Tet |
|  |  |  |  | Feces | 199f1 | *S. aureus* | yes | IV | Ery, Fox, Gen, Pen |
|  |  |  |  | Feces | 199f2 | *S. epidermidis* | yes | nt | Ery, Fox, Gen, Pen, SXT |
| **20** | M | 3 | M | Nasal | 200n | *S. aureus* | no | na | Pen, Tet |
|  |  |  |  | Feces | 200f | *S. aureus* | yes | III | Cli, Ery, Fox, Pen, Tet |
| **21** | S | 4 | F | Nasal | 201n1 | *S. aureus* | no | na | Pen |
|  |  |  |  | Nasal | 201n2 | *S. epidermidis* | no | na | Pen, Tet |
|  |  |  |  | Feces | 201f | *S. aureus* | yes | IV | Cli, Ery, Fox, Pen |
| **22** | S | 5 | M | Nasal | 202n | *S. aureus* | yes | IV | Fox, Pen |
|  |  |  |  | Feces | 202f1 | *S. aureus* | no | na | Pen |
|  |  |  |  | Feces | 202f2 | *S. epidermidis* | yes | V | Ery, Fox, Pen, SXT |
| **23** | MO | 2 | M | Nasal | 203n | *S. aureus* | yes | IV | Fox, Pen |
|  |  |  |  | Feces | 203f1 | *S. aureus* | yes | IV | Ery, Fox, Pen |
|  |  |  |  | Feces | 203f2 | *S. haemolyticus* | no | na | Ery, Pen |
| **24** | S | 2 | F | Nasal | 204n | *S. aureus* | yes | IV | Fox, Pen |
|  |  |  |  | Feces | 204f1 | *S. aureus* | yes | IV | Fox, Pen |
|  |  |  |  | Feces | 204f2 | *S. haemolyticus* | no | na | Pen |
| **25** | S | 6 | F | Nasal | 205n1 | *S. aureus* | no | na | Ery |
|  |  |  |  | Nasal | 205n2 | *S. epidermidis* | no | na | Gen, Pen, Tet |
|  |  |  |  | Feces | 205f | *S. haemolyticus* | yes | nd | Ery, Fox, Pen |
| **26** | MO | 3 | F | Nasal | 206n1 | *S. aureus* | no | na | Pen |
|  |  |  |  | Nasal | 206n2 | *S. haemolyticus* | no | na | Ery, Pen |
|  |  |  |  | Feces | 206f | *S. aureus* | no | na | Pen |
| **27** | S | 2 | M | Nasal | 207n | *S. epidermidis* | no | na | Ery, Pen, SXT |
|  |  |  |  | Feces | 207f | *S. sciuri* | no | na | Pen |
| **28** | M | 2 | F | Nasal | 208n | *S. epidermidis* | no | na | Pen |
|  |  |  |  | Feces | 208f1 | *S. aureus* | no | na | Pen |
|  |  |  |  | Feces | 208f2 | *S. haemolyticus* | no | na | Ery, Pen |
| **29** | M | 2 | M | Nasal | 209n | *S. saprophyticus* | no | na | Tet |
|  |  |  |  | Feces | 209f | *S. saprophyticus* | no | na | Ery |
| **30** | M | 2 | M | Nasal | 2010n | *S. epidermidis* | no | na | Pen |
|  |  |  |  | Feces | 210f1 | *S. aureus* | no | na | Cli, Ery, Pen |
|  |  |  |  | Feces | 210f2 | *S. epidermidis* | no | na | Pen |
| **31** | MO | 2 | M | Nasal | 211n | *S. saprophyticus* | yes | nd | Ery, Fox, Pen |
|  |  |  |  | Feces | 211f | *S. warneri* | yes | nd | Ery, Pen |
| **32** | MO | 5 | M | Nasal | 212n | *S. aureus* | yes | IV | Ery, Fox, Pen |
|  |  |  |  | Feces | 212f | *S. aureus* | yes | IV | Ery, Fox Pen |
| **33** | M | 5 | F | Nasal | 213n1 | *S. aureus* | no | na | Cli, Ery, Gen, Pen |
|  |  |  |  | Nasal | 213n2 | *S. epidermidis* | yes | V | Ery, Fox, Pen, Tet |
|  |  |  |  | Feces | 213f | *S. aureus* | no | na | Cli, Ery, Gen, Pen |
| **34** | MO | 10 | F | Nasal | 214n | *S. aureus* | yes | IV | Fox, Mup, Pen |
|  |  |  |  | Feces | 214f | *S. aureus* | yes | IV | Fox, Mup, Pen |
| **35** | M | 10 | M | Nasal | 215n | *S. aureus* | no | na | Cli, Pen |
|  |  |  |  | Feces | 215f1 | *S. aureus* | no | na | Cli, Ery, Pen |
|  |  |  |  | Feces | 215f2 | *S. epidermidis* | no | na | Cli, Ery, Pen, SXT, Tet |
| **36** | MO | 4 | M | Nasal | 216n | *S. aureus* | yes | IV | Ery, Fox, Pen |
|  |  |  |  | Feces | 216f | *S. aureus* | no | na | Pen |
| **37** | MO | 6 | F | Nasal | 217n1 | *S. aureus* | no | na | Cli, Ery, Gen |
|  |  |  |  | Nasal | 217n2 | *S. epidermidis* | yes | IV | Cip, Cli, Ery, Fox, Gen, Pen, STX |
|  |  |  |  | Feces | 217f1 | *S. aureus* | no | na | Cli, Ery, Gen, Pen |
|  |  |  |  | Feces | 217f2 | *S. epidermidis* | yes | IV | Fox, Gen, Pen |
| **38** | MO | 6 | F | Nasal | 218n1 | *S. aureus* | no | na | Pen |
|  |  |  |  | Nasal | 218n2 | *S. epidermidis* | yes | V | Ery, Fox, Pen, SXT, Tet |
|  |  |  |  | Feces | 218f | *S. aureus* | yes | IV | Ery, Fox, Pen |
| **39** | MO | 7 | M | Nasal | 219n | *S. aureus* | yes | V | Ery, Fox, Pen |
|  |  |  |  | Feces | 219f | *S. aureus* | yes | IV | Fox, Pen |
| **40** | MO | 8 | M | Nasal | 220n1 | *S. aureus* | no | na | Pen |
|  |  |  |  | Nasal | 220n2 | *S. epidermidis* | yes | V | Fox, Pen, SXT |
| **41** | MO | 4 | F | Nasal | 221n1* | *S. aureus* | yes | IV | Fox, Pen |
|  |  |  |  | Nasal | 221n2 | *S. hominis* | no | na | ** |
|  |  |  |  | Feces | 221f1 | *S. aureus* | no | na | ** |
|  |  |  |  | Feces | 221f2 | *S. epidermidis* | no | na | Pen |
| **42** | M | 4 | M | Nasal | 222n2 | *S. hominis* | yes | nd | Ery, Fox, Pen |
|  |  |  |  | Feces | 222f1 | *S. haemolyticus* | yes | nd | Ery, Fox, Pen |
|  |  |  |  | Feces | 222f2 | *S. epidermidis* | yes | V | Fox, Pen, SXT |
| **43** | S | 7 | F | Nasal | 223n1 | *S. aureus* | no | na | Pen |
|  |  |  |  | Nasal | 223n2 | *S. epidermidis* | yes | V | Fox, Pen, Tet |
|  |  |  |  | Feces | 223f1 | *S. cohnii* | no | na | Cli, Ery, Pen |
|  |  |  |  | Feces | 223f2 | *S. epidermidis* | yes | V | Ery, Fox, Pen, SXT, Tet |
| **44** | MO | 8 | F | Nasal | 224n1 | *S. aureus* | no | na | Ery, Gen, Pen |
|  |  |  |  | Nasal | 224n2 | *S. epidermidis* | yes | IV | Ery, Fox, Pen, SXT |
|  |  |  |  | Feces | 224f | *S. aureus* | yes | IV | Fox, Pen |
| **45** | S | 8 | F | Nasal | 225n* | *S. aureus* | yes | IV | Fox, Gen, Pen |
|  |  |  |  | Feces | 225f1* | *S. aureus* | yes | IV | Fox, Gen, Pen |
|  |  |  |  | Feces | 225f2 | *S. epidermidis* | yes | nt | Cli, Fox, Gen, Pen, Tet |
| **46** | MO | 3 | M | Nasal | 226n | *S. aureus* | no | na | Pen |
|  |  |  |  | Feces | 226f | *S. aureus* | no | na | Cli, Ery, Gen, Pen |
| **47** | S | 2 | M | Nasal | 227n | *S. aureus* | no | na | Pen |
|  |  |  |  | Feces | 227f | *S. aureus* | no | na | Ery, Pen |
| **48** | S | 5 | F | Nasal | 228n1* | *S. aureus* | yes | IV | Ery, Fox, Pen |
|  |  |  |  | Nasal | 228n2 | *S. epidermidis* | no | na | Ery |
|  |  |  |  | Feces | 228f1* | *S. aureus* | yes | IV | Ery, Fox, Pen |
|  |  |  |  | Feces | 228f2 | *S. epidermidis* | yes | V | Fox, Pen |
| **49** | S | 4 | F | Nasal | 229n1* | *S. aureus* | yes | IV | Ery Fox, Gen, Pen |
|  |  |  |  | Feces | 229f | *S. sciuri* | yes | nd | Cli, Fox, Pen |
| **50** | MO | 6 | M | Nasal | 230n1 | *S. aureus* | no | na | Pen |
|  |  |  |  | Nasal | 230n2 | *S. epidermidis* | yes | V | Ery, Fox, Pen, SXT |
|  |  |  |  | Feces | 230f* | *S. aureus* | no | na | Pen, Tet |
| **51** | MO | 9 | M | Nasal | 231n1 | *S. aureus* | no | na | Cli, Ery, Pen |
|  |  |  |  | Feces | 231f1 | *S. aureus* | no | na | Cli, Ery, Pen |
|  |  |  |  | Feces | 231f2 | CoNS | yes | nd | Cip, Fox, Pen, SXT |
| **52** | MO | 3 | M | Nasal | 232n1 | *S. aureus* | no | na | Cli, Ery, Pen |
|  |  |  |  | Feces | 232f1 | *S. saprophyticus* | no | na | Pen |
|  |  |  |  | Feces | 232f2 | *S. epidermidis* | no | na | Ery, Gen, Pen, SXT |
| **53** | S | 7 | M | Nasal | 233n1* | *S. aureus* | no | na | Pen, SXT |
|  |  |  |  | Feces | 233f1* | *S. aureus* | no | na | Pen, SXT |
|  |  |  |  | Feces | 233f2 | *S. epidermidis* | yes | IV | Fox, Pen |
| **54** | MO | 8 | F | Nasal | 234n1 | *S. aureus* | yes | IV | Fox, Pen |
|  |  |  |  | Nasal | 234n2 | CoNS | no | na | Cli, Ery, Rif, Tet |
|  |  |  |  | Feces | 234f1 | *S. aureus* | yes | IV | Fox, Pen |
|  |  |  |  | Feces | 234f2 | *S. haemolyticus* | yes | nd | Fox, Pen |
|  |  |  |  | Feces | 234f3 | *S. epidermidis* | yes | nt | Fox, Pen, Tet |
| **55** | M | 5 | M | Feces | 235f1 | *S. aureus* | no | na | Cli, Ery, Pen |
| **CONTROL GROUP** | | | | | | | | | |
| **Individual number** | **SCORAD** | **Age**  **(years)** | **Gender** | **Clinical source** | **Isolate**  **number** | **Species** | **Methicillin**  **resistance** | **SCC*mec***  **type** | **Antimicrobial resistance profile** |
| **1** | na | 10 | F | Feces | 1f | nc | na | na | na |
| **2** | na | 6 | M | Feces | 2f2 | CoNS | no | na | nd |
|  |  |  |  | Feces | 2f3 | *S. psicifermentans* | no | na | nd |
| **3** | na | 6 | M | Feces | 3f2 | *S. aureus* | no | na | nd |
| **4** | na | 2 | F | Feces | 4f1 | *S. aureus* | no | na | nd |
|  |  |  |  | Feces | 4f3 | *S. hominis* | yes | nt | nd |
| **5** | na | 10 | F | Feces | 5f2 | *S. cohnii* | yes | III | nd |
|  |  |  |  | Feces | 5f3 | *S. epidermidis* | yes | nt | nd |
| **6** | na | 2 | M | Feces | 6f1 | *S. aureus* | no | na | nd |
|  |  |  |  | Feces | 6f2 | *S. haemolyticus* | yes | IV | nd |
| **7** | na | 5 | M | Feces | 7f1 | *S. sciuri* | no | no | nd |
| **8** | na | 8 | F | Feces | 8f | *S. haemolyticus* | yes | nt | nd |
|  |  |  |  | Feces | 8f2 | *S. epidermidis* | no | na | nd |
| **9** | na | 4 | M | Feces | 9f1 | *S. haemolyticus* | yes | V | nd |
|  |  |  |  | Feces | 9f2 | *S. epidermidis* | no | na | nd |

SCORAD – Scoring atopic dermatitis; SCC*mec* - Staphylococcal chromosome cassete *mec*; M – mild; MO – moderate; S – severe; F- female, M – male; no - negative; yes – positive; na – not aplicable; nd – not determined; nt – not tiplable; nc – not colonized by *Staphylococcus* spp. Cip – ciprofloxacin; Cli – clindamycin; Ery - erythromycin; Fox – cefoxitin; Gen – gentamycin; Mup – mupirocin; Pen – penicillin; Rif – rifampicin; SXT - trimethoprim-sulfamethoxazole; Tet – tetracycline; * - Panton-Valentine leukocidin (PVL) positive isolate; ** - isolates susceptible to all antimicrobials evaluated.
