# Supplementary material for: Atopic dermatitis pediatric patients show high rates of nasal and intestinal colonization by methicillin-resistant Staphylococcus aureus and coagulase-negative staphylococci
Source: BMC Microbiol. 2024 Jan 29;24:42. doi: 10.1186/s12866-023-03165-5 (PMC10823624; doi:10.1186/s12866-023-03165-5)
Supplement: Supplementary file 3 — Additional file 3: Supplementary Table 2. Antimicrobial susceptibility of 84 Staphylococcus aureus isolates recovered from atopic dermatitis patients. [file 12866_2023_3165_MOESM3_ESM.doc]

| **Antimicrobials** | **N (%) of resistant isolates** | | |
| --- | --- | --- | --- |
|  | MRSA  (n=36) | MSSA  (n=48) | Total (n=84) |
| **Clindamycin** | 4 (11.1) | 16 (33.3) | 20 (23.8) |
| **Erythromycin** | 14 (38.9) | 22 (45.8) | 36 (42.9) |
| **Gentamicin** | 6 (16.7) | 9 (18.8) | 15 (17.9) |
| **Penicillin** | 36 (100) | 44 (91.7) | 80 (95.2) |
| **Mupirocin** | 3 (8.3) | 0 (0) | 3 (3.6) |
| **Tetracycline** | 2 (5.6) | 3 (6.3) | 5 (6) |
| **Trimethoprim-sulfamethoxazole** | 0 (0) | 2 (4.2) | 2 (2.4) |

**Supplementary table 2: Antimicrobial susceptibility of 84 *Staphylococcus aureus* isolates recovered from atopic dermatitis patients**

MRSA – methicillin-resistant *S. aureus*; MSSA – methicillin-sensitive *S. aureus*; All isolates were susceptible to ciprofloxacin and rifampicin
