# Supplementary material for: Atopic dermatitis pediatric patients show high rates of nasal and intestinal colonization by methicillin-resistant Staphylococcus aureus and coagulase-negative staphylococci
Source: BMC Microbiol. 2024 Jan 29;24:42. doi: 10.1186/s12866-023-03165-5 (PMC10823624; doi:10.1186/s12866-023-03165-5)
Supplement: Supplementary file 4 — Additional file 4:Supplementary Table 3. Chao and Shanon indexes associated with 45 AD pediatric patients and nine evaluated controls. [file 12866_2023_3165_MOESM4_ESM.doc]

| **Supplementary table 03**: Chao and Shanon indexes associated with 45 AD pediatric patients and nine evaluated controls | | | |
| --- | --- | --- | --- |
| **Number** | **SCORAD** | **Chao index** | **Shannon index** |
| Patient  53 | S | 15 | 2,71 |
| 43 | S | 17 | 2,83 |
| 48 | S | 16 | 2,77 |
| 45 | S | 12 | 2,48 |
| 1 | S | 20 | 3,00 |
| 16 | S | 23 | 3,14 |
| 18 | S | 13 | 2,56 |
| 47 | S | 17 | 0,94 |
| 3 | S | 21 | 0,95 |
| 22 | S | 17 | 0,94 |
| 14 | S | 15 | 2,71 |
| 27 | S | 15 | 0,93 |
| 19 | S | 9 | 0,89 |
| 49 | S | 17 | 0,94 |
| 54 | MO | 24 | 3,18 |
| 40 | MO | 17 | 0,94 |
| 32 | MO | 17 | 0,94 |
| 37 | MO | 27 | 0,96 |
| 50 | MO | 27 | 0,96 |
| 8 | MO | 18 | 0,94 |
| 34 | MO | 18 | 0,94 |
| 46 | MO | 16 | 0,94 |
| 39 | MO | 24 | 0,96 |
| 31 | MO | 27 | 0,96 |
| 36 | MO | 27 | 0,96 |
| 41 | MO | 35 | 0,97 |
| 51 | MO | 31 | 0,97 |
| 37 | MO | 22 | 0,95 |
| 44 | MO | 16 | 0,94 |
| 52 | MO | 20 | 0,95 |
| 5 | MO | 24 | 0,96 |
| 10 | MO | 23 | 0,96 |
| 2 | MO | 19 | 0,95 |
| 6 | MO | 20 | 0,95 |
| 9 | MO | 22 | 0,95 |
| 17 | MO | 11 | 0,91 |
| 42 | M | 18 | 2,89 |
| 20 | M | 21 | 0,95 |
| 55 | M | 15 | 0,93 |
| 30 | M | 19 | 0,95 |
| 33 | M | 22 | 0,95 |
| 4 | M | 16 | 0,94 |
| 13 | M | 21 | 0,95 |
| 15 | M | 13 | 0,92 |
| 28 | M | 17 | 0,94 |
| Control  8C | na | 17 | 0,94 |
| 9C | na | 16 | 0,94 |
| 3C | na | 20 | 0,95 |
| 5C | na | 20 | 0,95 |
| 6C | na | 17 | 0,94 |
| 1C | na | 17 | 0,94 |
| 4C | na | 18 | 0,94 |
| 2C | na | 17 | 0,94 |
| 7C | na | 11 | 0,91 |

SCORAD – Scoring atopic dermatitis; S- severe; MO-moderate; M-mild; na – not aplicable
